# Singleton exome sequencing of 90 fetuses with ultrasound anomalies revealing novel disease-causing variants and genotype–phenotype correlations

**DOI:** 10.1038/s41431-021-01012-7

**Published:** 2022-01-01

**Authors:** Mateja Smogavec, Maria Gerykova Bujalkova, Reinhard Lehner, Jürgen Neesen, Jana Behunova, Gülen Yerlikaya-Schatten, Theresa Reischer, Reinhard Altmann, Denisa Weis, Hans-Christoph Duba, Franco Laccone

**Affiliations:** 1 Institute of Medical Genetics, Medical University of Vienna, Vienna, Austria; 2 Division of Obstetrics and Feto-Maternal Medicine, Department of Obstetrics and Gynaecology, Medical University of Vienna, Vienna, Austria; 3 Department of Prenatal Medicine, Kepler University Hospital, School of Medicine, Johannes Kepler University, Linz, Austria; 4 Department of Medical Genetics, Kepler University Hospital, School of Medicine, Johannes Kepler University, Linz, Austria

**Keywords:** Genetics research, Disease genetics

## Abstract

Exome sequencing has been increasingly implemented in prenatal genetic testing for fetuses with morphological abnormalities but normal rapid aneuploidy detection and microarray analysis. We present a retrospective study of 90 fetuses with different abnormal ultrasound findings, in which we employed the singleton exome sequencing (sES; 75 fetuses) or to a lesser extent (15 fetuses) a multigene panel analysis of 6713 genes as a primary tool for the detection of monogenic diseases. The detection rate of pathogenic or likely pathogenic variants in this study was 34.4%. The highest diagnostic rate of 56% was in fetuses with multiple anomalies, followed by cases with skeletal or renal abnormalities (diagnostic rate of 50%, respectively). We report 20 novel disease-causing variants in different known disease-associated genes and new genotype–phenotype associations for the genes *KMT2D, MN1, CDK10,* and *EXOC3L2*. Based on our data, we postulate that sES of fetal index cases with a concurrent sampling of parental probes for targeted testing of the origin of detected fetal variants could be a suitable tool to obtain reliable and rapid prenatal results, particularly in situations where a trio analysis is not possible.

## Introduction

Structural fetal abnormalities are detected by prenatal ultrasound in ~3–4% of pregnancies [1], and 8.4–18.2% of these cases are caused by an abnormal karyotype [2, 3]. Microarray analysis detects causal submicroscopic rearrangements in an additional 6.0–8.2% of cases [3–5]. The introduction of exome sequencing into prenatal diagnostics in recent years extended the diagnostic yield by 8.5–50% [6–9]. The most recent prenatal exome-sequencing studies suggest an increasing transition from proband exome analysis to trio exome analysis [9–11]. Along with the increased genetic detection rate, prenatal whole-exome sequencing (WES) can also reveal new and/or unexpected associations between fetal phenotypes and identified variants, considerably extending the spectrum of prenatal manifestations of disease-causing variants in specific genes. Despite the ongoing discussions regarding indications for exome sequencing in a prenatal setting [12–14], the challenges of interpreting variants of unknown significance or of *de novo* variants in potential candidate genes, and strategies for managing secondary findings, this method has already become an important diagnostic tool in pregnancies with fetal abnormalities.

## Methods

### Patient selection

In this retrospective study, we included 90 fetuses with a normal Rapid Aneuploidy Detection (RAD) using Fluorescence in situ hybridization analysis and microarray analysis results, which was referred to as a genetic analysis from April 2015 to July 2020 as a clinical service. The cases included in this study have been selected of all consecutive fetuses with normal RAD and microarray testing undergoing prenatal genetic testing by referring gynecologists according to their best practice based on clinical assessment of fetal ultrasound phenotype suspicious of monogenic cause. Fetal samples were obtained by chorionic villus sampling (*n* = 29) and amniocentesis (*n* = 61). The gestational age at the time of testing ranged from 11 + 1 to 31 + 4 weeks, with the majority of fetuses being tested after the 20th week of pregnancy (*n* = 60 cases; 66.7%) and the rest of the fetuses (*n* = 30 cases; 33.3%) being tested before the 20th week of pregnancy with a peak in the 13th week of pregnancy. The fetuses showed various major and/or minor ultrasound abnormalities, including increased nuchal translucency (>3.5 mm), and lacked a previously detected causal aberration. When possible, parental blood samples were concurrently obtained for DNA extraction. If the parental samples for variant origin testing were immediately available, the collection-to-reporting turnaround time was ~3 weeks for all confirmatory methods used for detected variations as well as parental tests.

The 90 fetal samples sent to our laboratory for genetic analyses with the indication of fetal malformation received firstly a RAD analysis and secondly an array CGH. If both analyses showed a negative result, we proceeded to perform an sES (singleton exome sequencing) or in the first analyzed cases a multiple gene panels enriched for 6713 genes associated with Mendelian disorders. The reason for performing a singleton analysis was mostly due to unapproved costs of medical insurance for a trio analysis. Recently, we increasingly perform trio WES as the financial covering has improved.

### Exome-sequencing and bioinformatics analysis

Fetal DNA extraction was performed according to standard protocols. For 15 fetal samples, multigene panel testing was enriched for 6713 genes associated with Mendelian disorders by means of the TruSight One Expanded Sequencing Panel Kit, and for 75 fetal samples, enrichment for whole-exome analysis was performed with the TruSeq Exome Kit, both from Illumina (San Diego, California, USA). DNA fragments were paired-end sequenced on an *Illumina NextSeq500* system. We performed sES in 86 fetuses and trio analysis including parental samples in four fetuses.

The obtained sequencing reads were aligned to the NCBI human genome assembly (hg19) using the Burrows Wheeler Alignment Tool. Variant calling (HaplotypeCaller) was performed according to GATK best practice guidelines (available at https://gatk.broadinstitute.org/hc/en-us) for calling single-nucleotide variants, insertions, and deletions. The evaluation of the called variants was performed using VarSeq software from Golden Helix® (Bozeman, Montana, USA). The exome-wide average read coverage was 75, whereas that of the multigene panel was 110.

Variants were filtered based on minor allele frequency (MAF) using our in-house database including data from >1000 whole exomes and published disease-causing variants. Variants present as heterozygous in more than five cases or homozygous in more than three cases in the internal database were filtered out, followed by filtering based on the MAF listed in the Genome Aggregation Database (gnomAD) (MAF ≤ 0.5). The remaining variants were filtered for genes associated with HPO terms based on fetal malformations with predefined criteria, namely, read depth of >9, the current variant allele frequency of >0.35, and Phred scale base quality score >99. In a second step, the cutoff for the variant allele frequency was lowered to 0.19 to allow the detection of possible mosaicism. If this analysis did not reveal any possibly pathogenic or pathogenic variant, the data analysis was extended to the whole exome, where also non-OMIM disease-associated genes were analyzed.

We routinely used six prediction tools for independent assessments of the potential pathogenicity of filtered missense variants (SIFT, Polyphen2 HVAR, MutationTaster, MutationAssessor, FATHMM, FATHMM MKL Coding), and variants with at least four predictions as damaging/pathogenic/likely pathogenic were selected. Loss-of-function variants were independently considered for further analysis. All variants were assessed for their interpretation in the ClinVar database.

Reported variants were classified based on the ACMG guidelines [15]. Among diagnostic variants, we reported likely pathogenic and pathogenic variants (Table 1), in addition, we also reported variants of uncertain significance. Among the secondary findings, we reported only pathogenic or likely pathogenic variants (Table 2). All reported fetal variants were confirmed by Sanger sequencing. Where available, targeted testing of parental DNA to examine the origin of variants detected in fetal DNA was performed by Sanger sequencing. Pathogenic and likely pathogenic variants have been submitted to ClinVar with accession numbers from SCV001519055–SCV001519082.

**Table 1:** Fetal phenotypes and identified pathogenic and likely pathogenic variants in our cohort.

No.	Fetal phenotype	Gene	Variant	Reference sequence	Zygosity/inheritance	ACMG classification/criteria**	Disorder	Novel/known variant	ClinVar ID
1	NT > 3,5 mm, microgenia, mesomelia, syndactyly of fingers	*SF3B4*	c.763_781del, p.(Pro255Cysfs*59)	NM_005850.4	het, AD, unknown	5 PVS1/PM2-M/PP3-S	Acrofacial dysostosis 1, Nager type	Novel	SCV001519055
2	Hydrocephalus (2nd pregnancy with the same malformations)	*MPDZ*	c.4171C>T, p.(Arg1391Ter)	NM_001330637.1	comp het, AR	5	Hydrocephalus, congenital, 2, w/wo brain or eye anomalies	Known	VCV000667381
			c.5125_5126ins, p.(Tyr1709Cysfs*5)			5 PVS1/PM2-M/PP3-S		Novel	SCV001519056
3*	Bilateral renal agenesia	*ANOS1*	c.774del, p.(Trp258Cysfs*9)	NM_000216.3	hemi, XL, mat inherited	5	Hypogonadotropic hypogonadism 1 w/wo anosmia (Kallmann syndrome 1)	Known	SCV001519057
4*	Left-sided diaphragmatic hernia, ascites, pleural efussion, susp. duplex kidneys	*PTPN11*	c.218C>T, p.(Thr73Ile)	NM_002834.4	het, AD, *de novo*	5	Noonan syndrome 1	Known	VCV000013334
5	Shortened limbs, multiple fractures	*COL1A2*	c.1352G>A, p.(Gly451Asp)	NM_000089.3	het, AD, *de novo*	5 PP3-S/PS2-S/PM2-M/PP2-S	Osteogenesis imperfecta	Novel	SCV001519058
6*	NT > 7 mm, heart defect, multicystic dysplastic kidneys, shortened long bones, bilateral clubfoot, abnormal skull configuration	*COL27A1*	c.4519C>T, p.(Arg1507Ter)	NM_032888.3	hom, AR	5 PVS1/PM2-M/PP3-S	Steel syndrome	Novel	SCV001519059
		*PKD1*	c.3984G>C, p.(Trp1328Cys)	NM_001009944.2	hom, AD	4 PM1-M/PM2-M/PP2-S/PP3-S	Polycystic kidney disease 1	Novel	SCV001519060
7*	Fetal hydrops	*GUSB*	c.1084_1110del, p.(Asp362_Phe370del)	NM_000181.3	comp het, AR	4	Mucopolysaccharidosis VII	Known	SCV001519061
			c.1651C>T, p.(Gln551Ter)			5 PVS1/PM2-M/PP3-S		Novel	SCV001519062
8*	Skeletal dysplasia, IUGR (length of long bones and fetal weight < 3rd percentile, small thorax)	*CUL7*	c.5022_5025del, p.(Cys1674Trpfs*44)	NM_001168370.1	hom, AR	5 PVS1/PM2-M/PP3-S	3-M syndrome 1	Novel	SCV001519063
9*	Agenesis of corpus callosum, cerebellar hypoplasia, colpocephaly, transposition of great vessels, micropenis, fingers closed in fists, asymmetric shortened legs und malformed feet (2nd pregnancy with the same malformations)	*COG6*	c.511C>T, p.(Arg171Ter)	NM_020751.2	hom, AR	5	Congenital disorder of glycosylation, type IIl/Shaheen syndrome	Known	VCV000493007
10	Skeletal dysplasia, shortened long bones, bilateral clubfoot	*SLC26A2*	c.1020_1022del, p.(Val341del)	NM_000112.3	comp het, AR	4	Diastrophic dysplasia/Achondrogenesis 1B/	Known	VCV000065558
			c.1957T>A, p.(Cys653Ser)			5	Atelosteogenesis, type II	Known	VCV000004098
11	Skeletal dysplasia, shortened long bones	*SLC26A2*	c.749A>T, p.(Asp250Val)	NM_000112.3	comp het, AR	4 PM1-M/PM2-M/PM3-M/PP2-S/PP3-S	Diastrophic dysplasia/Achondrogenesis 1B/Atelosteogenesis, type II	Novel	SCV001519064
			c.835C>T, p.(Arg279Trp)			5		Known	VCV000004089
12*	Polycystic kidneys, polyhydramnios	*HNF1B*	c.494G>A, p.(Arg165His)	NM_000458.3	het, AD, *de novo*	5	Renal cysts and diabetes syndrome	Known	VCV000012647
13	Agenesis of corpus callosum, hydronephrosis, bilateral clubfoot, abnormal hand position	*KAT6B*	c.3903del, p.(Ser1303Valfs*31)	NM_012330.3	het, AD, *de novo*	4 PVS1/PS2-S/PM2-M	Genitopatellar syndrome/SBBYSS syndrome	Novel	SCV001519065
14^a^	Multicystic, dysplastic enlarged kidneys, extreme oligohydramnios	*KMT2D*	c.5642_5644+1del, p.(?)	NM_003482.3	het, AD, *de novo*	5 PVS1/PS2-S/PM2-M/PP3-S	Kabuki syndrome 1	Novel	SCV001519066
15	Polycystic kidneys, dextrocardia	*BBS2*	c.263del, p.(Gly88Alafs*6)	NM_031885.3	hom, AR	5	Bardet-Biedl syndrome 2	Known	VCV000217434
16	Thanatophoric dysplasia	*COL2A1*	c.2798G>A, p.(Gly933Glu)	NM_001844.4	het, AD, *de novo*	5 PS2-S/PM1-M/PM2-M/PP2-S/PP3-S	Platyspondylic lethal skeletal dysplasia, Torrance type/Achondrogenesis 2	Novel	SCV001519067
17	Brain malformation, bilateral clubfoot, pericardial effusion	*VANGL1*	c.838del, p.(Leu280Ter)	NM_138959.2	het, AD, *de novo*	5 PVS1/PS2-S/PM2-M/PP3-S	Caudal regression syndrome/Neural tube defects, susceptibility to	Novel	SCV001519068
18^b^	Shortened long bones (< 3 percentille)	*MN1*	c.3555C>A, p.(Cys1185Ter)	NM_002430.2	het, AD, *de novo*	5 PVS1/PS2-S/PM2-M/PP3-S	MN1-associated syndrome, CEBALID syndrome	Novel	SCV001519069
19#	NT > 3.5 mm, hydronephrosis, polycystic kidney, clubfoot, macrosomia, twin pregnancy, only one fetus with malformations	*GPC3*	c.175+1G>T, p.(?)	NM_004484.3	hemi, XL, mat inherited	5 PVS1/PM2-M/PP3-S	Simpson-Golabi-Behmel syndrome, type 1	Novel	SCV001519070
20	Microcephaly, agenesis of the right kidney	*NBN*	c.657_661del, p.(Lys219Asnfs*16)	NM_002485.4	hom, AR	5	Nijmegen breakage syndrome	Known	VCV000006940
21	Brain malformations, cardiomegaly (2nd pregnancy with the same malformations)	*TREX1*	c.236_243dup, p.(Ser82Leufs*9)	NM_033629.5	hom, AR	5	Aicardi-Goutieres syndrome 1	Known	SCV001519071
22^c^	Suspected fetal ciliopathy, cerebellar vermis hypoplasia, hyperechogenic enlarged kidneys, oligohydramnios (3rd pregnancy with similar malformations)	*EXOC3L2*	c.(90+1_91-1)_(404+1_405-1)del, p.(?)	NM_138568.3	hom, AR	5 PVS1	No OMIM disease association Publications: Meckel-Gruber-like syndrome	Novel	SCV001519072
23	Lissencephaly	*PEX1*	c.1587+1G>A, p.(?)	NM_000466.2	comp het, AR	5	Peroxisome biogenesis disorder 1 A/1B	Known	VCV000371701
			c.2875C>T, p.(Arg959Ter)			5		Known	VCV000371716
24	Fetal hydrops, mild ventriculomegaly, microstomia, short fingers, syndyctyly of all fingers on the one hand side, heart malformation, shortend long bones, bilateral curved femur bones	*FRAS1*	c.4259G>A, p.(Trp1420Ter)	NM_025074.6	comp het, AR	5 PVS1/PM2-M/PP3-S	Fraser syndrome 1	Novel	SCV001519073
			c.6433C>T, p.(Arg2145Ter)			5 PVS1/PM2-M/PP3-S		Novel	SCV001519074
25	Suspected brain and brain vessel malformations, further abnormalities not specified	*PTEN*	c.131G>A, p.(Gly44Asp)	NM_000314.6	het, AD, mat inherited	5	Cowden syndrome 1/Macrocephaly and autism syndrome	Known	VCV000427582
		*PTPN11*	c.922A>G, p.(Asn308Asp)	NM_002834.4	het, AD, *de novo*	5	Noonan syndrome 1	Known	VCV000013326
26^d^	Fetal hydrops, hydrocephalus, multicystic, dysplastic kidneys, lung hypoplasia, cardiomyopathy, retrognathia	*CDK10*	c.608+1G>A, p.(?)	NM_052988.4	hom, AR	5	Al Kaissi syndrome	Known	VCV000440757
27	Complex heart defect	*KMT2D*	c.11320C>T, p.(Gln3774Ter)	NM_003482.3	het, AD, *de novo*	5 PVS1-S/PS2-S/PM2-M/PP3-S	Kabuki syndrome 1	Novel	SCV001519075
28	Suspected skeletal dysplasia, shortened long bones, bilateral curved femurs, malformed thorax with bowed rips	*COL1A1*	c.1201G>A, p.(Gly401Ser)	NM_000088.3	het, AD, *de novo*	5	Osteogenesis imperfecta	Known	VCV000425596
29	Agenesis of corpus callosum	*DCC*	c.3073C>T, p.(Arg1025Ter)	NM_005215.3	het, AD, mat inherited	5 PVS1/PM2-M/PP3-S	Mirror movements 1 and/or agenesis of the corpus callosum	Novel	SCV001519076
30	Skeletal dysplasia	*COL1A1*	c.1930G>A, p.(Gly644Ser)	NM_000088.3	het, AD, *de novo*	5	Osteogenesis imperfecta, type II or III or IV	Known	VCV000392573
31	Multiple malformations with contraction of distal muscles of the hands, bilateral clubfoot, minimal skin edema	*NEB*	c.24871-1G>C, p.(?)	NM_001271208.1	hom, AR	5 PVS1/PM2-M/PP3-S	Nemaline myopathy 2	Novel	SCV001519077

*Tested via multigene panel encompassing 6713 genes.

**ACMG criteria are listed only for novel variants.

Cases denoted with superscripts (a, b, c, d) are further delineated in text.

*NT* nuchal translucency, *IUGR* intrauterine growth retardation, *AD* autosomal dominant inheritance, *AR* autosomal recessive inheritance, *XL* X-linked inheritance, *Hom* homozygous, *Het* heterozygous, *Hemi* hemizygous, *Comp het* compound het, proven trans phase of alleles. ClinVar: https://www.ncbi.nlm.nih.gov/clinvar/; ACMG variant classification [15] (5 = pathogenic, 4 = likely pathogenic, 3 = variant of unknown significance, 2 = likely benign, 1 = benign); variants are described according to HGVS nomenclature.

^#^Published separately in *Clin. Pract*. 2021, *11*(1), 75–80; 10.3390/clinpract11010012.

ACMG criterion applied:

PVS1: Null variant (nonsense, frameshift, canonical ±1 or 2 splice sites, initiation codon, single or multiexon deletion) in a gene where LOF is a known mechanism of disease.

PS2-S: *De novo* (both maternity and paternity confirmed) in a patient with the disease and no family history, used at a strong level.

PM1-M: Located in a mutational hot spot and/or critical and well-established functional domain without benign variation, used at a moderate level.

PM2-M: Absent from controls in gnomAD database, used at a moderate level.

PM3-M: For recessive disorders, detected in trans with a pathogenic variant, used at a moderate level.

PP2-S: Missense variant in a gene that has a low rate of benign missense variation and in which missense variants are a common mechanism of disease, used at supporting level.

PP3-S: Multiple lines of computational evidence support a deleterious effect on the gene or gene product, used at the supporting level.

There are no corresponding identifiers to patient numbers.

**Table 2:** Reported secondary findings in our cohort.

Case no.	Fetal phenotype	Gene and reference sequence	Identified variant	Disorder	Disorder inheritance, variant zygosity and origin	ACMG classification/criteria**	Novel/known variant	ClinVar ID
6*	NT > 7 mm, heart defect, multicystic dysplastic kidneys, shortened long bones, bilateral clubfoot, abnormal skull configuration	*SBDS* NM_016038.2	c.258+2T>C, p.(?)	Shwachman-Diamond syndrome	AR, hom, mother also homozygous, father heterozygous (parents are consanguineous)	5	Known	VCV000003196.14
8*	Skeletal dysplasia, IUGR (length of long bones and fetal weight < 3rd percentile, small thorax)	*KMT2D* NM_003482.3	c.5468-1G>A, p.(?)	Kabuki syndrome 1	AD, het, maternally inherited	5 PVS1/PM2-M/PP3-S	Novel	SCV001519078
20	Microcephaly, agenesis of right kidney	*BRCA2* NM_000059.3	c.7350_7354del, p.(Asn2450Lysfs*2)	Breast-ovarian cancer, familial, 2	AD, het, paternally inherited	5 PP5-VS/PVS1/PM2-M	Known	VCV000254601.2
		*DUOX2* NM_014080.4	c.605_621del, p.(Gln202Argfs*93)	Thyroid dyshormonogenesis 6	AR, hom	5	Known	SCV001519079

In all listed fetuses a diagnostic variant of underlying malformation has been identified.

*Tested with a panel encompassing 6713 genes.

*NT* nuchal translucency, *IUGR* intrauterine growth retardation, *AD* autosomal-dominant inheritance, *AR* autosomal recessive inheritance, *Hom* homozygous, *Het* heterozygous, ClinVar: https://www.ncbi.nlm.nih.gov/clinvar/.

**ACMG criteria are listed only for novel variants, ACMG variant classification [15] (5 = pathogenic, 4 = likely pathogenic, 3 = variant of unknown significance, 2 = likely benign, 1 = benign); variants are described according to HGVS nomenclature.

There are no corresponding identifiers to patient numbers.

ACMG criterion applied:

PVS1: Null variant (nonsense, frameshift, canonical ±1 or 2 splice sites, initiation codon, single or multiexon deletion) in a gene where LOF is a known mechanism of disease.

PM2-M: Absent from controls in gnomAD database, used at a moderate level.

PP3-S: Multiple lines of computational evidence support a deleterious effect on the gene or gene product, used at the supporting level.

PP5-VS: Reputable source recently reports variant as pathogenic, but the evidence is not available to the laboratory to perform an independent evaluation, used at a very strong level.

In addition, a copy number variation (CNV) analysis was performed for all analyzed samples comparing the calculated coverage of each sequenced sample to the already existing coverage data, obtained from BAM-files, for all previously analyzed in-house samples. This analysis was also done by a supported module from VarSeq within the VarSeq software from Golden Helix®. The reported fetal CNVs were confirmed by qPCR (multiexonic deletion in *EXOC3L2*).

## Results

### Fetal phenotypes

A large proportion of the fetuses showed either central nervous system (*n* = 28; 31.1%) or multiple structural anomalies (*n* = 25; 27.8%), followed by skeletal abnormalities (*n* = 16; 17.8%), increased nuchal translucency or fetal hydrops (*n* = 13; 14.4%) and renal abnormalities (*n* = 6; 6.7%). Two fetuses had an isolated complex heart defect. Fetuses with malformations in ≥2 organ systems were defined as having multiple anomalies.

### Diagnostic outcomes

**Fig. 1:**
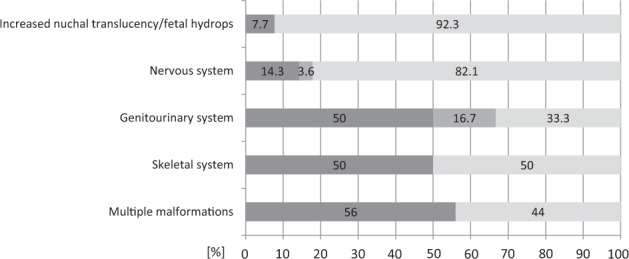
Molecular detection rates based on organ system involvement. Diagnostic rates are shown for fetuses with malformations in a specific organ system with identified pathogenic or likely pathogenic variants (dark gray), variants of unknown significance (VUS; medium gray), and no identified diagnostic variant (light gray). “Multiple malformations” indicates the involvement of ≥2 organ systems. Examples of isolated nervous system malformations: hydrocephalus, agenesis of the corpus callosum, cerebellar hypoplasia, lissencephaly; of the genitourinary system: renal agenesis, polycystic kidneys; of the skeletal system: multiple fractures, shortened limbs, intrauterine growth retardation (IUGR).

We observed an overall diagnostic yield of 34.4% (31 of 90, Table 1). In 3.3% (3 of 90) of the fetuses, we detected variants of uncertain significance. The diagnostic rate varied considerably within different phenotypic subgroups, and the greatest yield was reached in fetuses with multiple anomalies (56.0%; 14 of 25), followed by skeletal abnormalities (50.0%; 8 of 16) and renal abnormalities (50.0%; 3 of 6). A lower diagnostic rate was found in those with central nervous system abnormalities (14.3%; 4 of 28) and increased nuchal translucency or fetal hydrops (7.7%; 1 of 13) (Fig. 1). In one fetus with an isolated complex heart defect, we detected a heterozygous *de novo* pathogenic variant in the *KMT2D* gene (1 of 2).

In 45.1% (14 of 31) of the cases, we detected biallelic disease-causing variants for diseases inherited in an autosomal recessive fashion, and in 45.1% (14 of 31) of the fetuses, we detected *de novo* disease-causing variants or autosomal dominant inherited disorders. In one fetus, the origin of the autosomal dominant disease-causing variant in the *SF3B4* gene could not be defined because parental DNA was not available. Another two fetuses carried maternally inherited variants for X-linked disorders in the *GPC3* and *ANOS1* genes. Two fetuses carried two diagnostic variants each (*COL27A1* and *PKD1* in one fetus and *PTPN11* and *PTEN* in the second fetus).

Pathogenic and likely pathogenic variants as well as the fetal phenotypes are listed in Table 1.

### Further delineation of known phenotypes and unexpected findings

Here, we report new possible phenotypic associations and further delineation of prenatal features in three known disease-causing genes, *KMT2D, MN1,* and *CDK10*, as well as in a candidate disease-causing gene, *EXOC3L2*.

A fetus diagnosed with multicystic and dysplastic enlarged kidneys and severe oligohydramnios had a novel variant c.5642_5644+1del in *KMT2D* of *de novo* origin, detected by sES (case 14 in Table 1).

**Fig. 2:**
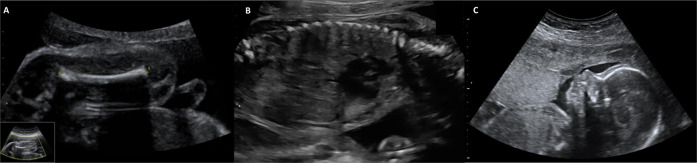
Ultrasound of the fetus with *de novoMN1* variant. The fetus showed in the 21st gestational week-long bone length under the 3rd percentile (**A**: femur, 29,3 mm) without any other skeletal abnormalities. During the further course of pregnancy, there have been no other malformation or abnormalities of the skeleton identified (thorax (**B**) and profile (**C**) considered as normal; 21st gestational week).

A fetus with sonographic signs of shortened long bones in the 23rd week of pregnancy (the length of all proximal long bones under the 3rd percentile) and a single umbilical artery confirmed by fetal MRI and no further abnormalities, had a novel nonsense variant in *MN1* of *de novo* origin (c.3555C>A, p.Cys1185Ter) (case 18 in Table 1 and Fig. 2).

**Fig. 3:**
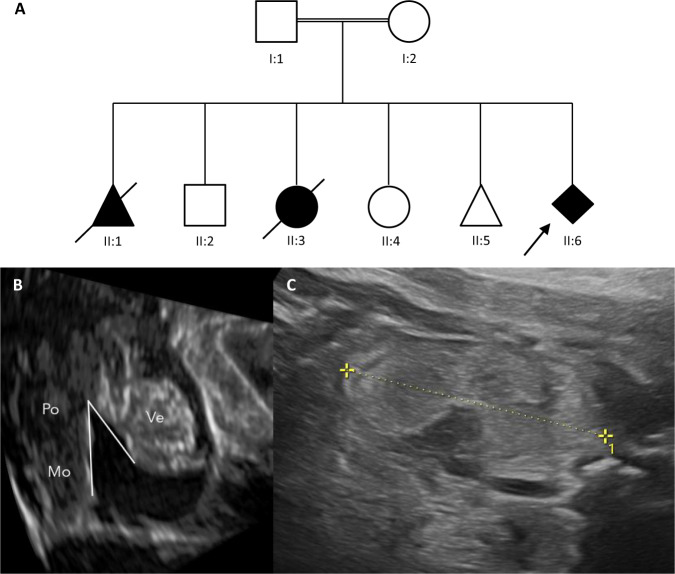
Pedigree of the consanguineous couple carrying a heterozygous deletion of exons 3, 4, and 5 in *EXOC3L2* and ultrasound findings of the fetus with *EXOC3L2* deletion in homozygous state. Both parents (I:1 and I:2) carry a heterozygous deletion of exons 3, 4, and 5 in *EXOC3L2*, which has been detected in the fetus from the last pregnancy (II:6) in a homozygous state (**A**). A male fetus in the first pregnancy (II:1) showed renal dysgenesis. The pregnancy has been interrupted in the 22nd gestational week. A healthy boy has been born from the 2nd pregnancy (II:2). In the 3rd pregnancy, an anhydramnios has developed and a girl has been born spontaneously in the 25th gestational week with a Dandy-Walker malformation and an encephalocele, who died shortly after the birth (II: 3). From the 4th pregnancy, a healthy girl has been born in the 39th gestational week (II:4). The 5th pregnancy ended in an abortion in the 15th gestational week (II:5). The fetus of the last pregnancy (II:6, index case) with a homozygous deletion in *EXOC3L2* showed in the 30 + 5 gestational week lung hypoplasia, hypoplastic vermis cerebelli (**B**: Ve – Vermis, Po – Pons, Mo – Medulla oblongata; cc 15,8 mm (<5. perc), ap 11,4 mm (25. perc.), brainstem-vermis angle: 38,2°) and hyperechogenic, enlarged kidneys (**C**).

A fetus of consanguineous parents showed cerebellar vermis hypoplasia, enlarged hyperechogenic kidneys, oligohydramnios, and lung hypoplasia in the 24th week of pregnancy. One previous pregnancy of the same couple was terminated because of a similar severe phenotype and in another pregnancy, with anhydramnios a girl was born in the 25th week with Dandy-Walker malformation and encephalocele, who died shortly afterward. Furthermore, the couple had one abortion and has two healthy children. For the current pregnancy, a trio whole-exome analysis was performed, and homozygous deletion of exons 3–5 in *EXOC3L2* was detected by CNV analysis. The parents were both heterozygous carriers of this deletion (case 22 in Table 1 and Fig. 3).

**Fig. 4:**
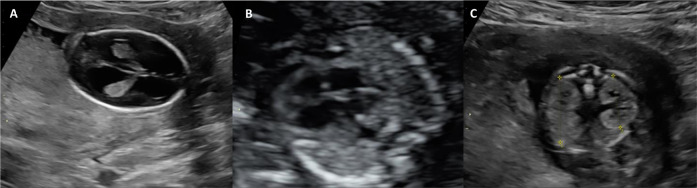
Ultrasound of the fetus with homozygous *CDK10* variant. At 16th gestational week, the fetus manifested several abnormalities in different organ systems: widened lateral (>10 mm) and 3rd brain ventricles (**A**); cardiomegaly and overrotation of the heart to the left (**B**); enlarged and hyperechogenic kidneys (**C**).

In a fetus diagnosed with fetal hydrops, multicystic dysplastic kidneys, cardiomyopathy, lung hypoplasia, retrognathia, and hydrocephalus in the 18th week of pregnancy, we performed an sES that showed a known homozygous disease-causing variant of the canonical donor splice site (c.608 + 1 G > A) in the *CDK10* gene. The parents were both heterozygous carriers of this disease-causing variant (case 26 in Table 1 and Fig. 4).

### Cases with two diagnostic variants

Two fetuses in our cohort carried two diagnostic variants each. In case 6, a fetus with nuchal transparency >7 mm, a heart defect, multicystic dysplastic kidneys, shortened long bones, bilateral clubfoot, and an abnormal skull configuration, we detected a homozygous nonsense *COL27A1* variant as well as a homozygous missense variant in *PKD1*. The parents were both heterozygous carriers of both variants. The *COL27A1* variant is suggested to be responsible for the skeletal phenotypic features, whereas the homozygous variant in *PKD1* is considered to be a hypomorphic dominant allele causing features of autosomal recessive polycystic kidney disease [16]. In case 25, we identified a known *PTPN11* disease-causing variant of *de novo* origin as well as a known disease-causing variant in the *PTEN* gene inherited from a previously undiagnosed mother. The 34 years old mother did not show any manifestations of Cowden syndrome and her family history regarding *PTEN*-associated tumors was unremarkable.

### Cases with potentially diagnostic variants of uncertain significance

In addition to 31 fetuses with confirmed genetic diagnosis, three fetuses had inconclusive results. A fetus with lissencephaly carried a heterozygous novel missense variant in *TUBA1A* (NM_006009.3: c.680T>A, p.(Leu227Gln); SCV001519080). In a fetus with an isolated complex heart defect, we identified heterozygous missense variants in two genes (*ASXL2*: NM_018263.4:c.2847T>G, p.(Asn949Lys); VCV000931398.2 and *MYH6*: NM_002471.3: c.2134C>G, p.(Pro712Ala); SCV001519081). All three of these variants were predicted as damaging or possibly damaging with five of six prediction tools and were classified as variants of uncertain significance (VUS). No paternal DNA was available for either fetus; thus, a definitive assessment of the variants was not possible. Finally, a fetus with polycystic kidney disease carried two missense variants in *PKD1* (NM_001009944.2: c.11872G>C, p.(Ala3958Pro); SCV001519082, paternal and c.9499A>T, p.(Ile3167Phe); VCV000440135, maternal) in a compound heterozygous state. The father, who was a carrier of one of the missense variants, as well as paternal aunt and paternal grandmother, were all known to have polycystic renal disease without an identified genetic cause. The mother carried the other missense variant, however, has not been known to have polycystic kidney disease at the time of genetic testing. Further family members were not available for segregation analysis.

### Secondary findings

Every data set was proofed for secondary findings. We detected and reported four secondary findings in three fetuses (detection rate of 3.3%; including two variants in one fetus). These variants are listed in Table 2. In all three of these fetuses, the main cause of the anomalies was detected. A paternally inherited loss-of-function variant in *BRCA2* in case 20 was the only variant in a medically actionable gene identified [17, 18]. A secondary finding in this fetus was a homozygous frameshift variant in *DUOX2* leading to congenital hypothyroidism. Case 6 carried a known splicing variant in the *SBDS* gene in a homozygous state, in addition to the two diagnostic variants in *COL27A1* and *PKD1* discussed above. The testing of the consanguineous parents revealed a heterozygous carrier status in the father and, surprisingly, homozygosity of the *SBDS* variant in the mother, thus confirming the diagnosis of Shwachman-Diamond syndrome (SDS) in the fetus and the mother. The phenotypic spectrum of SDS has been shown to be rather broad, and asymptomatic individuals have also been described [19]. The contribution of the *SBDS* variant to some of the fetal phenotypic features (especially shortened long bones) cannot be fully excluded. However, given the familial setting, we consider it as a secondary finding. In case 8, which had a diagnostic variant in *CUL7*, we also identified a maternally inherited novel splicing variant in the *KMT2D* gene as a secondary finding. The phenotyping of the mother for potential symptoms of Kabuki syndrome was unfortunately not possible.

## Discussion

### Diagnostic yield, prioritization strategy, and new genotype–phenotype associations

In our retrospective study of a broad spectrum of fetal structural abnormalities in 90 prenatal cases referred to our institute and investigated primarily with sES, we observed an overall diagnostic yield of 34.4%.

Several studies have shown the diagnostic utility of prenatal WES in fetuses with different ultrasound abnormalities [20]. The detection yield varies widely according to the selection criteria, number of genes investigated within WES analysis, variant prioritization, singleton versus trio analysis, number of investigated cases, and parental consanguinity. The first studies with a smaller number of fetal cases (<15) initially showed a diagnostic yield of above 40% [21, 22]; recent studies with 103 and 105 prenatal cases reported lower diagnostic rates, of 20.6 and 19% [11, 23], and another two studies with 234 and 610 prenatal cases recorded even lower diagnostic yields of 8.5% and 10%, respectively [9, 10]. One feature common to all the published data on prenatal WES is a higher detection yield in fetuses with multiple structural anomalies and skeletal anomalies than in fetuses with other types of structural or isolated anomalies [11, 24]. This trend is also observed in our data (diagnostic rate with multiple malformations of 56.0% and with skeletal anomalies of 52.9%). However, the two prospective studies with the largest number of investigated fetuses and a fetal-parent trio exome strategy have a significantly lower diagnostic yield, as in our retrospectively assessed cohort [9, 10]. This may be in part due to the conservative approach of variant classification used by Petrovski and coworkers, which may lead to the underreporting of variants. Lord et al. selected only a subset of genes associated with developmental disorders, and the study criteria aimed to include a particular spectrum of phenotypes so that the number of fetuses with any specific phenotype was capped at ~20% of the ongoing total. Avoiding the pre-filtering of the analyzed gene set according to OMIM phenotypic associations may be of particular importance as shown in our case 22. This case had a biallelic loss-of-function variant of the *EXOC3L2* gene, which is not included in the virtual gene panel adopted by Lord et al. and has not yet been associated with any OMIM listed disease; this case likely would have been missed by both previously mentioned approaches. *EXOC3L2*, however, has been proposed as a novel candidate gene for a lethal ciliopathy phenotype that resembles Meckel-Gruber syndrome [25]. A similar prenatal phenotype with the brain (Dandy-Walker) and renal malformations (fetal enlarged kidneys) as well as extreme oligohydramnios and biallelic variants in *EXOC3L2*, as in our case, has been described previously [26, 27]. The overlapping clinical features, as well as supporting data from mouse models, are strong evidence of an association between biallelic loss-of-function in the *EXOC3L2* gene and the development of ciliopathy (http://www.informatics.jax.org/allele/MGI:5548646) [28]. Paradoxically, the use of trio exome analysis can also lead to a lower detection rate as sES analysis. Trio exome analysis is indeed the most effective and straightforward approach in rapid detection of *de novo* variants as well as defining the mono- or biallelic position of two variants. However, depending on the filtering strategy used, inherited pathogenic variants could be missed, as in our case 25 with a maternally inherited variant of the *PTEN* gene or in our case 29 with a maternally inherited variant in the *DCC* gene. Familial, previous unknown variants are an important factor to consider in genetic counseling for further family planning.

Our high detection rate of 34.4% for pathogenic and/or likely pathogenic variants is comparable to the detection rate reported by Normand et al. [8] in fetuses with at least one structural anomaly; although the high detection rate cannot be fully explained, there are several aspects that may contribute to this outcome. The prioritization and assessment strategy is likely one element of pivotal importance. We used a hypothesis-driven strategy for prioritization of the variants that included different filters for dominant versus recessively inherited diseases. An additional reason for the high detection rate may be a selection of fetuses with major structural malformations at the highly specialized feto-maternal unit, although this selection was based on general clinician expertise and not on any predefined criteria. Our detection rate is, however, in line with the diagnostic rate of 36.7% in an exome study carried out during the first 100 days of life in neonates with severe disease [29]. The similarity of our prenatal yield and the reported postnatal detection rate is encouraging and may be considered to confirm the validity of our bioinformatics approach and final assessment strategy. Each institution and laboratory develops its own pipeline, which depends on the number of investigated genes, bioinformatics assessment, and final assessment strategy. This could also explain the large variability in diagnostic yields and turnaround times from receiving the sample to the final report.

A major challenge of prenatal WES is the limited knowledge and developmental phase-specific detection of the fetal phenotypes associated with known disease-causing genes and with non-OMIM disease-associated genes. We detected a heterozygous *de novo* variant in the *KMT2D* gene in fetal case 14 with multicystic and dysplastic enlarged kidneys and extreme oligohydramnios. Among patients with *KMT2D* disease-causing variants, 30–40% develop urinary tract abnormalities, with hydronephrosis being the most frequent [30, 31]. Renal dysplasia is deemed to be part of the spectrum of Kabuki syndrome (OMIM #147920) in ~15–21% of patients [32, 33]. To our knowledge, the association of *KMT2D* variants with prenatally diagnosed multicystic or polycystic enlarged kidneys is a very rare finding [34]. However, their possible role in cystic kidney formation can be hypothesized because KMT2D protein is known to be expressed in the metanephros of mice (http://www.informatics.jax.org/) as well as in the human kidney (https://www.proteinatlas.org/ENSG00000167548-KMT2D/tissue). A second particular challenge was case 18, with a *de novo* MN1 truncation variant at its C-terminus. The *MN1* gene was only recently associated with CEBALID syndrome (Craniofacial defects, dysmorphic ears, structural brain abnormalities, expressive language delay, and impaired intellectual development; OMIM #618774) [35, 36]. The published cases with CEBALID syndrome carried *de novo* C-terminal *MN1* truncation variants that were suggested to act in a dominant-negative or gain-of-function manner. To date, no case with shortening of the proximal long bones associated with *MN1* variant has been described. Because of the *de novo* origin, the loss-of-function variant, and the possibility that nonsense-mediated mRNA decay is triggered in our fetal case, we propose it to be probably associated with the fetal skeletal phenotype in our case. This could be a novel or a fetus-specific presentation of the variant in the *MN1* gene, but we cannot exclude an independent, undetected genetic cause of the underlying shortening of long bones. Both above-mentioned examples highlight the challenge of fetal genotype–phenotype correlations, which are still in an early stage. Owing to the developing nature and time-limited clinical assessment of prenatal malformations, there are inherent difficulties in phenotype-guided genetic analysis and prompt detection of a meaningful genotype via WES.

### Variants of unknown significance, secondary findings, and missed variants

Additional issues in prenatal genetic diagnostics are the presence of VUS as well as the detection of secondary pathogenic findings. The possibility of detecting secondary findings in actionable genes should be discussed with the parents during genetic counseling prior to the analysis, although the stress and anxiety of the situation may lead to a misunderstanding of the consequences of their decision. In three fetal cases, we detected and reported four secondary findings (detection rate of 3.3%; including two variants in one fetus). Even more challenging are cases with the presence of VUS without a clear genetic etiology of the malformation and cases in which supplementary investigations are required, prolonging the period of uncertainty.

A further concern in prenatal exome analysis is the unknown proportion of missed, unidentified, or assessed-as-non-pathogenic variants. As a recent example, an unreported homozygous nonsense variant in the *ARMC9* gene was identified in a family with a second affected pregnancy with the same phenotype before adequate information in the medical literature was available to make a genetic diagnosis [10]. In such cases, the knowledge of an association between a specific gene and a phenotype at the time of analysis may be a relevant issue; it is also possible that the bioinformatics pipeline will filter out a causative variant in a known disease-causing gene. This potential risk could hinder the more widespread addition or first-line use of exome analysis to the spectrum of routine prenatal diagnostics, as failing to identify the causative variant can have major legal consequences. In addition, a well-defined strategy assessing this aspect is necessary so that the involved clinicians can counsel the family appropriately.

### Study limitations

This study was based on coded data available from our internal clinical database. As such, we recognize several limitations. The first limitation of this study is its retrospective character with the inclusion of a subset of fetuses that were referred for prenatal sES after prior negative RAD and array CGH analysis based only on the clinician´s decision according to the assessment of the ultrasound phenotype. As a result, our cohort may represent a selected population of fetuses with malformations in which there was a higher probability of an underlying monogenic cause. This type of selection bias would tend to inflate the diagnostic yield. The second limitation of this study is our inability to access additional clinical information on all pregnancies in our cohort. As a result, we cannot indicate what percentage of pregnancies was terminated, how many children were born, and how was the phenotypic evolution after birth. We are also unable to access information or do a segregation analysis in the families that might have allowed us to confirm or refute a particular diagnosis. This further information may have allowed us to reclassify variants of unknown significance. This limitation could be minimized in a prospective study in which full access to all medical records would be possible.

In conclusion, our retrospective study confirms the validity of exome analysis as a prenatal diagnostic tool that offers families additional opportunities to end the diagnostic odyssey during pregnancy. A very important requirement before even starting the WES analysis is that the precise description of fetal ultrasound and/or even fetal MR results should be available to the genetic laboratory. With the improvement of bioinformatics tools for the detection of CNVs, exome or genome analyses could become the routine first-tier analyses after negative RAD results in prenatal diagnostic workflows.

## Data Availability

The data sets generated and/or analyzed during the current study are available from the corresponding author on reasonable request.
